# Intersexual allometry differences and ontogenetic shifts of coloration patterns in two aquatic turtles, *Graptemys oculifera* and *Graptemys flavimaculata*

**DOI:** 10.1002/ece3.1517

**Published:** 2015-05-20

**Authors:** Joshua R Ennen, Peter V Lindeman, Jeffrey E Lovich

**Affiliations:** 1 Tennessee Aquarium Conservation Institute201 Chestnut St., Chattanooga, Tennessee, 37402; 2 Department of Biology and Health Services, Edinboro University of Pennsylvania230 Scotland Rd., Edinboro, Pennsylvania, 16444; 3 Southwest Biological Science Center, U.S. Geological SurveyFlagstaff, Arizona

**Keywords:** Allometry, coloration, epiphenomenon, *Graptemys*, sexual niche partitioning, sexual selection

## Abstract

Coloration can play critical roles in a species' biology. The allometry of color patterns may be useful for elucidating the evolutionary mechanisms responsible for shaping the traits. We measured characteristics relating to eight aspects of color patterns from *Graptemys oculifera* and *G. flavimaculata* to investigate the allometric differences among male, female, and unsexed juvenile specimens. Additionally, we investigated ontogenetic shifts by incorporating the unsexed juveniles into the male and female datasets. In general, male color traits were isometric (i.e., color scaled with body size), while females and juvenile color traits were hypoallometric, growing in size more slowly than the increase in body size. When we included unsexed juveniles in our male and female datasets, our linear regression analyses found all relationships to be hypoallometric and our model selection analysis found support for nonlinear models describing the relationship between body size and color patterns, suggestive of an ontogenetic shift in coloration traits for both sexes at maturity. Although color is critical for many species' biology and therefore under strong selective pressure in many other species, our results are likely explained by an epiphenomenon related to the different selection pressures on body size and growth rates between juveniles and adults and less attributable to the evolution of color patterns themselves.

## Introduction

The evolution of coloration (i.e., pigmentation and pattern) is intrinsically linked to a species' specific ecology. For example, coloration is sometimes critical for effective camouflage, which conceals an individual from predator and prey (i.e., crypsis and ground pattern matching, Endler 1978, 1981, 1984; disruptive coloration, Cuthill et al. 2005). In ectothermic individuals, coloration can aid in thermoregulation through differences in basking behavior and performance between lighter and darker individuals (Luke 1989). Additionally, many species utilize their coloration for intraspecific (e.g., mate choice) and interspecific signaling purposes (e.g., species recognition, Losos 1985; Couldridge and Alexander 2002; aposematism, Mappes et al. 2005; and mimicry, Pasteur 1982; Pough 1988; Mallet and Joron 1999). The most commonly cited color-centric intraspecific signaling is mate choice (birds, Gray 1996; Kimball and Ligon 1999; Badyaev and Hill 2003; fish, Page 1983; Kodric-Brown 1998; and frogs, Bell and Zamudio 2012), which can lead to sexual dichromatism, that is, intersexual color differences. Sexual dichromatism can result from sexual or natural selection (Shine 1989), but sexual selection is the more commonly cited mechanism (Bell and Zamudio 2012). However, in rare cases, natural selection can produce sexual dichromatism if intersexual habitat preferences exist, where the sexes are under different selection pressures and sexual niche partitioning occurs (Heinsohn et al. 2005).

Many studies use allometry and relative amount of variation (i.e., coefficient of variation) of traits to elucidate the evolutionary mechanism (natural vs. sexual selection) responsible for shaping traits. Traits under sexual selection are often exaggerated (reviewed by Andersson 1994) and may display positive allometry (e.g., Alatalo et al. 1988; Møller 1991; Burkhardt et al. 1994; Bonduriansky 2006; but see Bonduriansky 2007) and higher coefficients of variation (Møller 1991; Alatalo et al. 1988), but positive allometry and higher coefficients of variation are not exclusively associated with traits under sexual selection in all taxa (Eberhard et al. 1998; Eberhard 2002; Bonduriansky 2007). Although there is not a consensus on the roles of natural and sexual selection in shaping allometry (see Bonduriansky 2007), most allometry studies investigate noncolor pattern traits (e.g., wings, appendages, and feathers). For example, in Bonduriansky's (2007) review, the author cited one allometry study investigating a color pattern trait (black color area) in *Poecilia reticulata* (Kelly et al. 2000), which displayed positive allometry. Therefore, there is a paucity of information pertaining to evolutionary mechanisms for shaping allometry of color patterns of organisms.

*Graptemys* is a highly patterned and colorful turtle genus restricted to North America. Species' patterns of coloration are informative for species identification and taxonomy (Cagle 1954; Lovich and McCoy 1992; Vogt 1993; Ennen et al. 2000a,b). However, little is known about color evolution within this genus and what is known suggests natural and sexual selection both influence coloration and patterns. In *Graptemys nigrinoda*, coloration and pattern are presumed to be related to camouflage under natural selection because this species exhibits clinal variation along the river continuum, where individuals inhabiting slower, more turbid water near the river mouth were darker than individuals in faster, clearer water (Ennen et al. 2014). Head patterns, in particular postorbital blotches, might be under sexual selection in *Graptemys geographica*, in which chroma, hue, and brightness of the postorbital blotch differ between males and females, creating sexual dichromatism (Bulté et al. 2013). Additionally, coloration and color patterns might be important in the courtship of *Graptemys* because males face females while displaying. In the *Graptemys pseudogeographica* complex, which displays temperature-dependent sex determination, Vogt (1993) reported that head patterns were influenced by incubation temperatures, thereby creating variable sexual dichromatism at hatching. Additionally, some *Graptemys* exhibit sexual niche partitioning (e.g., *G. flavimaculata*, Jones 1996; *G. versa*, Lindeman 2003), with males inhabiting areas closer to the bank than females, which could create differing selection pressure on pigmentation for camouflage.

The exact evolutionary mechanism shaping color patterns and sexual dichromatism in *Graptemys* is unclear. In this study, we investigate color pattern allometry in *Graptemys oculifera* and *Graptemys flavimaculata*. In particular, we compare the allometry among males, females, and juveniles to make inferences about the evolutionary mechanism (i.e., natural or sexual selection) influencing color patterns that could be further studied in turtles.

## Materials and Methods

### Data collection

We measured (i.e., length and width) characteristics relating to eight aspects of color pattern from 205 preserved specimens of two species of aquatic turtle, *Graptemys flavimaculata* (55 males, 26 females, 32 juveniles) and *G. oculifera* (44 males, 34 females, and 14 juveniles) to compare allometry between juveniles, males, and females (see Table S1 for museum catalog numbers). From each specimen, we recorded straight-line plastron length (PL) and eight measurements of line and blotch patterns from the head and forelimb, which included length (LPOB) and width (WPOB) of the postorbital blotch, width of the upper (NLU) and lower (NLL) yellow lines entering the orbit on the side of the head, length (LIOL) and width (WIOL) of the interorbital line, and width of yellow line on the forelimb leading to the second (WY2F) and fourth (WY4F) digit. Although museum collections are biased toward males, juveniles, and smaller females in turtles, our sample encompassed most of the body-size variation in *G. oculifera* and *G. flavimaculata* females (see Selman 2012; Lindeman 2013). We sexed turtles based on secondary sex characteristics, which develop in males of *Graptemys* species at different sizes (Cagle 1954; Lahanas 1982; Jones and Selman 2009; reviewed by Lindeman 2013). Therefore, we used a minimum carapace length of 72 mm for both species in this study as reported by Jones and Selman (2009) for *G. oculifera* and size ranges from Lindeman (2013) and Selman (2012) to determine sex.

### Statistical analysis

Before we could investigate the allometry and ontogenetic shifts of our eight color patterns, we needed to determine whether geography influenced color patterns. Similar to Ennen et al. (2014), we used cumulative drainage area (CDA) as a surrogate for geography at the point of collection. We conducted several linear regressions using CDA and each color patterns for each species' males, females, and juvenile (except for *G. oculifera* juveniles, which had a very low sample size of specimens with locality information). To account for size (PL) differences among individuals, we divided each color pattern value by PL to create color pattern ratios and then used an arcsine square root transformation. Additionally, we used a log-transformation for CDA. Because we only found one color pattern significantly (WY4F; *P* = 0.006) related to CDA for male *G. flavimaculata*, the geography of the specimens is not influencing color patterns within these species to a high degree. Therefore, we proceeded with analyzing our data in several ways to elucidate allometry and ontogenetic patterns. First, we analyzed the groups (i.e., male, female, and unsexed juvenile) separately using log–log linear regression to identify the relationship of each of the color pattern with PL for each species. For all significant regression tests, we categorized the relationship as isometric, hypoallometric (i.e., negative allometry), or hyperallometric (i.e., positive allometry) using slopes (i.e., isometric: *b *= 1; hypoallometric: *b *< 1; hyperallometric: *b *> 1) and 95% confidence intervals (CI). If the 95% CI bracketed a slope value of 1, then the relationship was considered isometric. If both lower and upper limits of the 95% CI were <1 or >1, then the relationship was considered significantly hypoallometric or hyperallometric, respectively.

Next, we created datasets that incorporated juveniles (referred to below as pooled) with mature adults of each sex for each species to investigate allometry through log–log regressions and ontogenetic shifts through model selection analysis and *R*^2^ value comparison. In model selection, we fitted different regression models (i.e., linear, exponential, and power) to the raw data and compared the best fit via Akaike Information Criterion corrected for sample size (AICc, Burnham and Anderson 2002). Models with ΔAIC_c_ values <2 were considered to have “substantial” support and thus the best models for the data (Burnham and Anderson 2002). If our model selection found support for a linear relationship, then no ontogenetic shift occurs in the color pattern trait because the relationship between color pattern and body size remained constant. However, if our model selection found substantial support for either the exponential or power model, then an ontogenetic shift does occur in the color pattern trait because the relationship between the color pattern and body size changes. We elected to pool unsexed juveniles in these analyses for several reasons. First, differences in allometry between males and females (without pooled juveniles) in our previous analysis with unpooled data could result from differences in statistical power associated with the large differences in range of body sizes between males and females. For example, males have narrow size ranges, so significant departure from isometry may be harder to identify than it is within the much wider range in body size of the females. Second, Gibbons and Lovich (1990) stated “natural selection should operate equally and in the same manner on both sexes, while they are juveniles with similar sizes and behaviors, i.e., prior to attainment of maturity”. Additionally, we elected to pool because we did not have permission to dissect specimens from the museums to determine sex and combining unsexed juveniles with adults has been used in numerous growth studies involving turtles (e.g., Frazer et al. 1990). All statistical tests were conducted in R (R Development Core Team 2013), and all measurements were recorded from the right side of the specimens by JRE.

## Results

### Color pattern ratios

Color pattern ratios of juveniles were greater than those of males and females in both species for the color patterns measured (Table1). In both species, males had greater color pattern ratios than females, except in LOPB. In general, *G. flavimaculata* had greater color pattern ratios than *G. oculifera* in head patterns (LOPB, WOPB, and WIOL).

**Table 1:** Mean color pattern ratios (i.e., pattern divided by plastron length) for eight color pattern traits in juvenile, males, and females for two species of *Graptemys*, *G. flavimaculata* and *G. oculifera*

Species/Sex	LOPB	WOPB	NLU	NLL	LIOL	WIOL	WY2F	WY4F
*Graptemys flavimaculata*								
Juvenile	0.060	0.093	0.021	0.032	0.174	0.030	0.029	0.036
Female	0.047	0.044	0.010	0.017	0.106	0.016	0.016	0.022
Male	0.046	0.056	0.012	0.022	0.123	0.020	0.020	0.025
*Graptemys oculifera*								
Juvenile	0.045	0.072	0.017	0.032	0.174	0.027	0.024	0.028
Female	0.036	0.038	0.009	0.014	0.100	0.012	0.017	0.016
Male	0.034	0.045	0.010	0.019	0.134	0.015	0.020	0.019

### Allometry

For males of both species, all coloration characters that were significantly related to body size (all but WPOB in *G. flavimaculata*, only NLL and LIOL in *G. oculifera*) exhibited isometric relationships (Table2). In contrast, coloration characters that were significantly related to body size in females and juveniles exhibited a mix of hypoallometric (*N* = 16) and isometric (*N* = 5) relationships. In males, only WPOB was hypoallometric (both species), although it was not related to body size. Female specimens exhibited isometry in two characters for both species, while three characters were hypoallometric in *G. flavimaculata* and four were in *G. oculifera* (Table2). Females of both species exhibited isometry for LPOB, and each species exhibited isometry for different characters on the forelimb. Comparing the two species, females exhibited congruent results (hypoallometry) for NLL and LIOL but incongruent results for WY4F. Only two characters, NLL and LIOL, were significantly related to body size in both species and both sexes. For both NLL and LIOL, males of both species exhibited isometry while females of both species exhibited hypoallometry. All coloration characters that were significantly related to body size exhibited hypoallometry in juvenile specimens except for WOPB in *G. oculifera*, which was isometric. Six characters had significant relationships with body size and were significantly hypoallometric in *G. flavimaculata* juveniles, while there were only three such characters in *G. oculifera*. When juvenile specimens were included in male and female datasets (i.e., pooled data) in log–log regression analyses, all relationships for males and females were significantly hypoallometric (Table3). The disparity in body-size ranges between the sexes likely confounded our previous separate analyses of males and females using unpooled data.

**Table 2:** Allometric relationships of color pattern traits (log-transformed) with body size (log-transformed plastron length) in males and females of *Graptemys oculifera* and *Graptemys flavimaculata*. Asterisks indicate significant relationships

Species	Mean (mm)	CV	*r* ^2^	*P*	Y-Intercept	Slope	95% Confidence Interval		Allometry
							Lower	Upper	
*Graptemys flavimaculata*									
Male (*n* = 55)									
LOPB	3.73	0.16	0.18	0.00	−1.06	0.85 ± 0.24	0.36	1.34	Isometric^*^
WOPB	4.53	0.18	0.02	0.32	0.09	0.29 ± 0.29	−0.30	0.88	
NLU	0.95	0.21	0.10	0.02	−1.58	0.81 ± 0.33	0.15	1.48	Isometric^*^
NLL	1.74	0.16	0.19	0.00	−1.43	0.88 ± 0.25	0.38	1.37	Isometric^*^
LIOL	9.97	0.12	0.24	0.00	−0.31	0.68 ± 0.17	0.35	1.02	Isometric^*^
WIOL	1.64	0.19	0.09	0.02	−1.11	0.69 ± 0.29	0.10	1.28	Isometric^*^
WY2F	2.04	0.16	0.15	0.00	−1.51	0.95 ± 0.31	0.32	1.58	Isometric^*^
WY4F	1.94	0.20	0.10	0.02	−1.25	0.80 ± 0.33	0.14	1.45	Isometric^*^
Female (*n* = 26)									
LOPB	5.31	0.24	0.46	0.00	−1.01	0.84 ± 0.19	0.46	1.22	Isometric^*^
WOPB	4.89	0.35	0.03	0.43	0.01	0.32 ± 0.39	−0.49	1.12	
NLU	1.06	0.19	0.33	0.00	−1.07	0.53 ± 0.15	0.22	0.85	Hypoallometric^*^
NLL	1.89	0.22	0.30	0.00	−0.93	0.58 ± 0.18	0.21	0.96	Hypoallometric^*^
LIOL	11.82	0.12	0.77	0.00	−0.03	0.54 ± 0.06	0.41	0.66	Hypoallometric^*^
WIOL	1.78	0.25	0.05	0.27	−0.36	0.29 ± 0.26	−0.24	0.82	
WY2F	2.44	0.27	0.13	0.07	−0.60	0.47 ± 0.25	−0.04	0.98	
WY4F	2.68	0.38	0.59	0.00	−2.34	1.34 ± 0.23	0.87	1.81	Isometric^*^
Juveniles (*n* = 33)									
LOPB	2.57	0.22	0.39	0.00	−0.56	0.59 ± 0.13	0.32	0.86	Hypoallometric^*^
WOPB	3.89	0.25	0.00	1.00	0.58	0.0002 ± 0.2	−0.41	0.41	
NLU	0.90	0.15	0.16	0.02	−0.48	0.26 ± 0.1	0.04	0.49	Hypoallometric^*^
NLL	1.38	0.15	0.28	0.00	−0.44	0.35 ± 0.1	0.15	0.56	Hypoallometric^*^
LIOL	7.43	0.10	0.42	0.00	0.39	0.29 ± 0.06	0.17	0.42	Hypoallometric^*^
WIOL	1.29	0.37	0.04	0.24	−0.32	0.25 ± 0.2	−0.17	0.67	
WY2F	1.24	0.19	0.41	0.00	−0.80	0.54 ± 0.12	0.30	0.78	Hypoallometric^*^
WY4F	1.55	0.14	0.33	0.00	−0.39	0.35 ± 0.09	0.17	0.54	Hypoallometric^*^
*Graptemys oculifera*									
Male (*n* = 39)									
LOPB	2.66	0.27	0.01	0.52	−0.35	0.4 ± 0.62	−0.86	1.67	
WOPB	3.41	0.22	0.00	0.86	0.70	−0.09 ± 0.51	−1.13	0.94	
NLU	0.76	0.23	0.03	0.30	−1.20	0.57 ± 0.54	−0.52	1.66	
NLL	1.45	0.20	0.10	0.05	−1.79	1.03 ± 0.51	−0.01	2.07	Isometric^*^
LIOL	10.27	0.18	0.15	0.02	−0.86	0.99 ± 0.39	0.20	1.77	Isometric^*^
WIOL	1.19	0.33	0.09	0.07	−2.17	1.18 ± 0.63	−0.10	2.46	
WY2F	1.57	0.28	0.08	0.09	−1.56	0.92 ± 0.53	−0.15	2.00	
WY4F	1.50	0.24	0.04	0.25	−1.11	0.67 ± 0.58	−0.50	1.85	
Female (*n* = 32)									
LOPB	4.39	0.31	0.46	0.00	−1.14	0.84 ± 0.17	0.49	1.20	Isometric^*^
WOPB	4.57	0.29	0.28	0.00	−0.59	0.59 ± 0.18	0.23	0.95	Hypoallometric^*^
NLU	1.02	0.21	0.00	0.97	−0.01	0.01 ± 0.16	−0.32	0.33	
NLL	1.72	0.18	0.35	0.00	−0.66	0.42 ± 0.11	0.20	0.65	Hypoallometric^*^
LIOL	11.98	0.26	0.32	0.00	−0.18	0.60 ± 0.16	0.27	0.93	Hypoallometric^*^
WIOL	1.36	0.32	0.03	0.36	−0.25	0.18 ± 0.19	−0.21	0.58	
WY2F	2.10	0.27	0.64	0.00	−1.42	0.83 ± 0.12	0.59	1.06	Isometric^*^
WY4F	1.98	0.26	0.37	0.00	−0.92	0.58 ± 0.14	0.29	0.87	Hypoallometric^*^
Juveniles (*n* = 14)									
LOPB	1.90	0.24	0.28	0.05	−0.69	0.59 ± 0.27	0.00	1.18	
WOPB	3.06	0.29	0.40	0.01	−1.17	1.01 ± 0.36	0.23	1.79	Isometric^*^
NLU	0.68	0.14	0.13	0.21	−0.53	0.22 ± 0.17	−0.14	0.58	
NLL	1.30	0.17	0.00	0.93	0.07	0.02 ± 0.22	−0.47	0.51	
LIOL	7.33	0.15	0.61	0.00	−0.04	0.56 ± 0.13	0.27	0.84	Hypoallometric^*^
WIOL	1.13	0.50	0.00	0.83	−0.14	0.1 ± 0.47	−0.92	1.12	
WY2F	1.02	0.20	0.37	0.02	−0.87	0.54 ± 0.2	0.09	0.98	Hypoallometric^*^
WY4F	1.15	0.12	0.39	0.02	−0.55	0.37 ± 0.13	0.08	0.66	Hypoallometric^*^

**Table 3:** Allometric relationships of color pattern traits (log-transformed) with body size (log-transformed plastron length) for datasets that incorporated juvenile specimens with both male and female specimens. Asterisks indicate significant relationships

Species	*r* ^2^	*P*	Y-Intercept	Slope	95% Confidence Interval		Allometry
					Lower	Upper	
*Graptemys flavimaculata*							
Male (*n* = 88)							
LOPB	0.66	0.00	−0.60	0.61 ± 0.05	0.52	0.71	Hypoallometric^*^
WOPB	0.10	0.00	0.25	0.21 ± 0.07	0.08	0.34	Hypoallometric^*^
NLU	0.05	0.04	−0.25	0.12 ± 0.06	0.00	0.23	Hypoallometric^*^
NLL	0.45	0.00	−0.48	0.37 ± 0.04	0.29	0.46	Hypoallometric^*^
LIOL	0.73	0.00	0.14	0.45 ± 0.03	0.39	0.51	Hypoallometric^*^
WIOL	0.30	0.00	−0.57	0.41 ± 0.07	0.27	0.54	Hypoallometric^*^
WY2F	0.70	0.00	−1.17	0.78 ± 0.05	0.67	0.89	Hypoallometric^*^
WY4F	0.32	0.00	−0.37	0.34 ± 0.05	0.24	0.45	Hypoallometric^*^
Female (*n* = 58)							
LOPB	0.83	0.00	−0.81	0.74 ± 0.04	0.65	0.83	Hypoallometric^*^
WOPB	0.08	0.03	0.29	0.18 ± 0.08	0.02	0.34	Hypoallometric^*^
NLU	0.33	0.00	−0.38	0.20 ± 0.04	0.12	0.27	Hypoallometric^*^
NLL	0.56	0.00	−0.41	0.33 ± 0.04	0.25	0.41	Hypoallometric^*^
LIOL	0.92	0.00	0.11	0.47 ± 0.02	0.43	0.51	Hypoallometric^*^
WIOL	0.32	0.00	−0.45	0.34 ± 0.07	0.21	0.47	Hypoallometric^*^
WY2F	0.76	0.00	−0.99	0.66 ± 0.05	0.56	0.76	Hypoallometric^*^
WY4F	0.67	0.00	−0.72	0.55 ± 0.05	0.45	0.65	Hypoallometric^*^
*Graptemys oculifera*							
Male (*n* = 55)							
LOPB	0.29	0.00	−0.66	0.56 ± 0.12	0.32	0.80	Hypoallometric^*^
WOPB	0.12	0.01	−0.05	0.31 ± 0.11	0.08	0.53	Hypoallometric^*^
NLU	0.07	0.06	−0.49	0.19 ± 0.10	0.00	0.38	
NLL	0.07	0.05	−0.22	0.19 ± 0.10	0.00	0.39	Hypoallometric^*^
LIOL	0.56	0.00	−0.07	0.57 ± 0.07	0.43	0.71	Hypoallometric^*^
WIOL	0.03	0.20	−0.27	0.17 ± 0.14	−0.10	0.45	
WY2F	0.49	0.00	−1.10	0.68 ± 0.10	0.49	0.87	Hypoallometric^*^
WY4F	0.24	0.00	−0.63	0.42 ± 0.10	0.21	0.63	Hypoallometric^*^
Female (*n* = 45)							
LOPB	0.79	0.00	−0.99	0.77 ± 0.06	0.65	0.90	Hypoallometric^*^
WOPB	0.47	0.00	−0.27	0.44 ± 0.07	0.30	0.58	Hypoallometric^*^
NLU	0.42	0.00	−0.65	0.31 ± 0.06	0.20	0.42	Hypoallometric^*^
NLL	0.48	0.00	−0.34	0.27 ± 0.04	0.19	0.36	Hypoallometric^*^
LIOL	0.66	0.00	0.07	0.48 ± 0.05	0.38	0.59	Hypoallometric^*^
WIOL	0.14	0.01	−0.31	0.21 ± 0.08	0.05	0.37	Hypoallometric^*^
WY2F	0.85	0.00	−1.12	0.69 ± 0.04	0.60	0.77	Hypoallometric^*^
WY4F	0.73	0.00	−0.76	0.50 ± 0.05	0.41	0.60	Hypoallometric^*^

### Ontogenetic shifts

**Figure 1:**
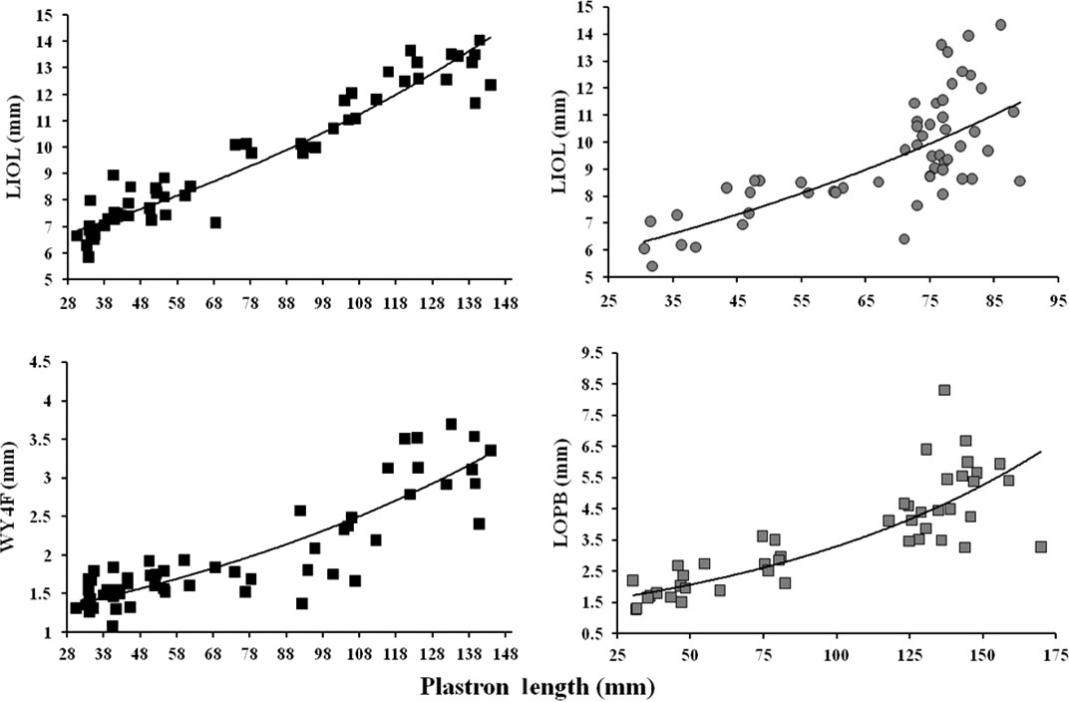
The best fitted models for the relationship between coloration patterns and body size (i.e., plastron length), in general, were nonlinear for *Graptemys flavimaculata* (black symbols) and *G. oculifera* (gray symbols) specimens. Square symbols represent females, while circles represent males.

We found that the best model for the majority (87.5%) of the coloration pattern versus body size relationships was nonlinear exponential and power models, that frequently had similar support based on AIC_c_ values (Table4), suggesting that an ontogenetic increase occurs in coloration patterns (Fig.1). Only NLU had support for a linear model in both sexes of both species; however, in three instances, a nonlinear model also had support. To further validate model selection, the *R*^2^ values gave similar results.

**Table 4:** The model selection results for male and female *Graptemys flavimaculata* and *G. oculifera* for each of the color patterns. The values in the table represent ΔAICc with *r*^2^ values in parentheses

Species/Sex/Model	Coloration patterns							
	LOPB	WOPB	NLU	NLL	LIOL	WIOL	WY2F	WY4F
*Graptemys flavimaculata*								
Male (*n* = 88)								
Linear	213.6 (0.61)	254.6 (0.1)	0.0 (0.06)	88.6 (0.45)	406.5 (0.67)	99.1 (0.2)	139.1 (0.58)	115.0 (0.3)
Exponential	0.8 (0.65)	0.0 (0.12)	5.3 (0.05)	0.0 (0.47)	0.0 (0.74)	0.0 (0.31)	0.0 (0.71)	0.0 (0.33)
Power	0.0 (0.66)	1.2 (0.1)	5.7 (0.05)	3.2 (0.45)	2.1 (0.73)	1.3 (0.3)	3.6 (0.7)	0.8 (0.32)
Female (*n* = 58)								
Linear	1614 (0.82)	166.5 (0.15)	0.6 (0.35)	64.5 (0.57)	252.5 (0.93)	64.8 (0.25)	97.1 (0.65)	127.6 (0.62)
Exponential	1.5 (0.83)	0.0 (0.1)	0.0 (0.35)	0.0 (0.58)	0.6 (0.91)	0.4 (0.31)	5.7 (0.73)	0.0 (0.73)
Power	0.0 (0.83)	0.9 (0.08)	2.1 (0.33)	2.7 (0.56)	0.0 (0.92)	0.0 (0.32)	0.0 (0.78)	11.5 (0.67)
*Graptemys oculifera*								
Male (*n* = 55)								
Linear	103.5 (0.24)	127.2 (0.07)	0 (0.08)	25.8 (0.11)	253.4 (0.5)	44 (0.01)	67.4 (0.33)	37.3 (0.25)
Exponential	0.1 (0.29)	1.3 (0.1)	31.3 (0.07)	0.0 (0.09)	0.0 (0.56)	0.0 (0.04)	0.0 (0.49)	0.0 (0.25)
Power	0.0 (0.29)	0.0 (0.12)	31.6 (0.07)	1.0 (0.07)	0.4 (0.56)	0.3 (0.03)	0.3 (0.49)	0.6 (0.24)
Female (*n* = 45)								
Linear	130.2 (0.68)	131.6 (0.38)	0.0 (0.32)	45.6 (0.48)	214.5 (0.63)	42.7 (0.07)	70.5 (0.76)	67.9 (0.62)
Exponential	3.1 (0.77)	2.6 (0.44)	5.3 (0.35)	0.0 (0.51)	1.3 (0.65)	0.6 (0.13)	0.0 (0.85)	2.3 (0.72)
Power	0.0 (0.79)	0.0 (0.47)	0.6 (0.42)	2.6 (0.48)	0.0 (0.66)	0.0 (0.14)	0.2 (0.85)	0.0 (0.73)

## Discussion

In general, color pattern allometry has been understudied relative to other traits (reviewed by Bonduriansky 2007) and no other study has investigated color pattern allometry in turtles. Only one species of 96 in Bonduriansky's (2007) review, the fish *Poecilia reticulata*, reported allometry of a color pattern trait (i.e., black color area). Interestingly, this color pattern trait was a sexual trait in males and was hyperallometric (Kelly et al. 2000). *Graptemys* species create a unique challenge for allometry analyses because of the extreme sexual size dimorphism, where females are often twice the length and several times the mass of males (reviewed by Lindeman 2013). This large disparity in intersexual body-size range could have contributed to the incongruence in allometry between the sexes in our analyses of pooled and unpooled datasets. For example, after combining the juvenile specimens within each sex and thus alleviating the intersexual body-size range disparity, all relationships for both sexes were hypoallometric, suggesting that the size of the lighter-colored features increased at a slower rate than body size.

Our model selection analyses, which fitted linear and curved lines to the pooled data set, and color pattern ratios found support for ontogenetic shifts in both sexes of both species. The majority of the color patterns had nonlinear relationships to body size, suggesting that the lighter-colored features grow at more rapid rates after maturity than in juveniles (but see below) but still more slowly than body size. Similarly, our color pattern ratios show a clear ontogenetic shift between juveniles and adults, but not, in general, between males and females (Table1). Ontogenetic shift in coloration has been reported for other *Graptemys* species and other turtle species. For example, Bulté et al. (2013) reported differences in brightness, hue, and chroma between adult male and adult female *Graptemys geographica,* but did not find coloration differences between small females and adult males. Bulté et al. (2013) found no support for hormonal levels (i.e., testosterone) influencing coloration (but see Lovich et al. 1990 and Garstka et al. 1991 on melanism) and speculated that dietary preferences could cause this phenomenon. Although there is considerable dietary overlap among small individuals and dietary niche partitioning between adult males and females in numerous *Graptemys* species (see Seigel and Brauman 1994 for *G. flavimaculata*; reviewed for the genus *Graptemys* by Lindeman 2000, 2013), no study has investigated the molecular components of the yellow pigmentation within *Graptemys* to determine whether the yellow is carotenoid based, which would be obtained via diet (Goodwin 1986).

Biological explanations for the color ontogenetic shifts are not fully understood in *Graptemys* largely because of the ecology of this genus is poorly studied (Lovich and Ennen 2013). Therefore, we provide several hypotheses and provide literature to support or refute each hypothesis. Four such hypotheses are predator deterrence, sexual niche partitioning, sexual selection, and by-product of growth rates. In other turtle species, ontogenetic shifts of plastral color patterns are known, in which hatchlings and juveniles possess brighter and more patterned plastrons than adults. Plastral coloration and patterns are suggested as predator deterrents in hatchlings and juveniles and function as an aposematic signal (see Semlitsch and Gibbons 1989; Britson and Gutzke 1993; Britson 1998). This phenomenon may explain ontogenetic shifts in *Graptemys* as well. For example, Folkerts and Mount (1969) reported the loss of plastral coloration patterns with size in *G. nigrinoda*. Although we did not measure plastral color patterns in our study, juveniles did possess greater relative amounts (lengths and widths) of lighter color patterns than adults (Table2). This trend could suggest juvenile color patterns play a role in deterring predators (i.e., aposematic coloration; see Britson and Gutzke 1993; Britson 1998) or in crypsis (see below).

Ontogenetic shifts in color patterns between juveniles and adult males and females could suggest individuals occupy different niches (i.e., sexual niche partitioning) related to sex and/or size, and therefore, have different color patterns for crypsis. Sexual niche partitioning is rarely applied to intersexual coloration difference explanations within a species (see Heinsohn et al. 2005; Bell and Zamudio 2012) and never has been applied to allometry. However, this hypothesis has limitations because it is only applicable to the juvenile/female ontogenetic color shifts, where there is clear habitat partitioning occurring (Lindeman 2013). Although males and females exhibit clear niche partitioning in several species (e.g., *G. geographica* – Pluto and Bellis 1986; *G. caglei* – Craig 1992; *G. flavimaculata* – Jones 1996; *G. versa* – Lindeman 2003), there is no evidence for niche partitioning between males and juveniles in *Graptemys*.

Under the sexual selection hypothesis, at maturity males allocate more resources to pigmentation relative to juveniles due its importance in courtship and mate choice in some species. However, no study has experimentally provided evidence of a sexually selected color trait in *Graptemys*, and sexual selection cannot explain the ontogenetic shift of coloration from juvenile to females as evident from the nonlinear relationships.

Finally, our results could have no adaptive significance and may be merely a by-product (i.e., epiphenomenon) of differing selection pressures on body size/growth rates for males, females, and juveniles (see Gould and Lewontin 1979). *Graptemys* species exhibit extreme sexual dimorphism, where females are larger than males (Lindeman 2013) and the strength and type of selection pressures (i.e., natural and sexual selection) on body size differs between the sexes (see Gibbons and Lovich 1990). Not only is selection pressure different for body size between males and females, it is also different for body growth rates between juveniles and adults (see Janzen et al. 2000). The differing selection pressure on body size and growth rates in *Graptemys* through time could influence pigmentation allometry greatly, because allometric patterns are ultimately a function of the “net selection experienced on trait size and body size, an ontogenetic resource-allocation trade-offs between these traits, and genetic constraints” (Bonduriansky 2007). Therefore, our nonlinear relationships could be attributed to changes in body growth rates through time – rapid growth of hatchlings/juveniles and slow growth rates after maturity – and less attributable to actual selection pressure (e.g., sexual selection and/or sexual niche partitioning) on the coloration pattern itself. Although sexual selection (for males only) and sexual niche partitioning (for females only) would be insignificant forces in juvenile color evolution, these mechanisms would become stronger and more significant after maturity when selection pressure on growth rates reduce. Therefore, the coloration patterns in *Graptemys* are most likely linked to multiple evolutionary mechanisms acting at different times and strengths.

## Supporting Information

**Table S1.** Museum catelog numbers for all measured specimens used in our analyses.
