# Supplementary material for: Intersexual allometry differences and ontogenetic shifts of coloration patterns in two aquatic turtles, Graptemys oculifera and Graptemys flavimaculata
Source: Ecol Evol. 2015 May 20;5(11):2296–305. doi: 10.1002/ece3.1517 (PMC4461428; doi:10.1002/ece3.1517)
Supplement: Supplementary file 1 [file ece30005-2296-sd1.pdf]

Supporting Information

Additional Supporting information may be found in the online version of this article:

Table S1.

Species	Museum Catelog Number
Graptemys oculifera	AUM: 5951-53, 5979, 9333, 14289, 23668, 25136-37, 25139; CM: 62156, 62158-59, 67468-72, 94882, 94888, 94915, 94927, 94931, 95034, 95049, 95060, 95557, 95641, 95666; LSUMZ: 7610, 12601, 53439, 80741-43, 85789, 86132, 86134, 86178; MMNS: 3280-81, 3729-33, 3752-54, 3756, 3874, 3995, 3997, 4001-02, 4004-05, 4007-08, 4010, 5535, 5639, 5953, 7610, 7681-84, 7686; TU: 21438-40, 21441-46, 21448-50, 21645, 21727-29, 21733, 21816, 21827, 21885
Graptemys flavimaculata	AUM: 5968, 5970-71, 5973, 6147, 6387, 8941-42, 8978, 9328-31, 9538, 9540-42, 9544, 10294, 10296, 13660-61, 23664; CM: 67437, 67447-51, 94958-59, 94969, 95353, 95558, 95574-76, 95876; MMNS: 1022, 1040, 1043, 1045, 1052-54, 1057, 1086, 1088-89, 1091, 1095, 1117-19, 3728, 4012, 5641, 5696-98, 5700-04, 5706-12, 5804, 10754; TU: 14752, 14757-60, 14762-63, 14766, 14774, 14779-85, 14799, 14804, 14806-08, 14809, 14818, 14826, 14832, 14845, 14850, 14858, 14862, 14865-66, 14868, 14870-71, 14873, 16546.30, 148665, 149221, and one specimen not labeled.
